# Quality of life in patients with locked-in syndrome: Evolution over a 6-year period

**DOI:** 10.1186/s13023-015-0304-z

**Published:** 2015-07-19

**Authors:** Marie-Christine Rousseau, Karine Baumstarck, Marine Alessandrini, Véronique Blandin, Thierry Billette de Villemeur, Pascal Auquier

**Affiliations:** Fédération des Hôpitaux de Polyhandicap et Multihandicap Hôpital San Salvadour, Assistance Publique Hôpitaux de Paris, BP 30 080, 83 407 Hyères, Cedex France; EA 3279 Self-Perceived Health Assessment Research Unit, School of Medicine, Aix Marseille Université, Marseille, France; Methodologic and Clinical Research Plateform, Assistance Publique Hôpitaux de Marseille, Marseille, France; French Association of Locked-in syndrome (ALIS), Paris, France; Fédération des Hôpitaux de polyhandicap et Multihandicap Hôpital Trousseau, Assistance Publique Hôpitaux de Paris, Paris, France

**Keywords:** Locked-in-syndrome, Quality of life, Anamnestic Comparative Self-Assessment, Determinants

## Abstract

**Background:**

Improved knowledge of the quality of life (QoL) of locked-in syndrome (LIS) patients have implications for managing their care, and assists clinicians in choosing the most appropriate interventions. We performed a survey of a population of LIS patients to describe the course of the QoL of LIS patients over a 6-year period and to determine the potential predictive factors of QoL changes over time.

**Method:**

This is a study performed over a 6-year period in patients with a LIS diagnosis. Questionnaires were sent in 2007 and 2013. The following data were recorded: i) sociodemographic data; ii) clinical data related to LIS, physical/handicap status, psychological status; iii) self-reported QoL: Anamnestic Comparative Self-Assessment (ACSA); iv) Integration in life: French Reintegration to Normal Living Index (RNLI).

**Results:**

Among the 67 patients included in 2007, 39 (58 %) patients returned their questionnaire in 2013. The LIS etiology was stroke in 51 individuals. The QoL of the patients was relatively satisfactory compared to populations in other severe conditions. Twenty-one (70 %) individuals reported a stable/improved QoL between 2007 and 2013. The physical/handicap statuses in 2007 and 2013 were not related to the QoL 6 years later, with the exception of one communication parameter: the individuals who used yes-no code reported significantly lower QoL levels than those who did not in 2013.

**Discussion:**

In opposition to a widespread opinion, LIS persons report a relatively satisfactory QoL level that stays stable over time, suggesting that life with LIS is worth living. Preservation of autonomy and communication may help them to live as normal life as possible.

## Background

Locked-in syndrome (LIS) is a neurologic condition characterized by the paralysis of all four limbs, anarthria, and lower cranial nerve paralysis that most often results from a brainstem lesion [1]. Although the mortality is high in the early stages of LIS (acute LIS) at 87 % within the first 4 months for LIS of vascular origin,[2] early rehabilitation and more effective nursing care have been reported to reduce mortality. Consequently, once an LIS patient becomes medically stable, despite the persistent and severe physical impairments, life expectancy can be significantly improved using appropriate medical care [3]. The life expectancies of stable LIS patients may be very long; 83 % of patients live 10 years, and 40 % live 20 years [4, 5]. The issue of the “quality” of this life also remains an important challenge. As with chronic diseases in general,[6] improved knowledge of the quality of life (QoL) and the determinants of the QoL of LIS patients have implications for managing their care, including considerations of ethical issues, and assists clinicians in choosing the most appropriate interventions. Therefore, QoL assessments are becoming increasingly important regarding evaluations of disease progression and the treatment and management of care in chronic diseases. Large international health agencies (e.g., the European Medicines Agency and the US Food and the Drug Administration) encourage the use of QoL assessments, and recommendations for QoL assessments are available [7, 8].

Several studies have previously explored the QoL of LIS patients [5, 9–14] and surprisingly have shown that the QoL of LIS patients is often in the same range as that of healthy individuals. Despite their extreme physical impairment, a number of LIS patients maintain a good QoL. In severely disabled people, some determinants of QoL have been previously identified; these factors include disability/handicap status [15], medical devices [13], social/familial support [14], depression [16]. Sociodemographic variables, such as gender and level of education, that traditionally impact the QoL of the individuals, were not really found as QoL predictors in these specific populations. But, the majority of these findings are based on single cross-sectional research studies that cannot show direction of association and may not provide definite information about cause and effect relationships. Evidence regarding the evolution of QoL over long durations and predictors of mid- and long-term QoL are lacking. These considerations incited us to conduct a survey of a population of LIS patients to describe the course of the QoL of LIS patients over a 6-year period and to determine the potential contribution of sociodemographic and clinical factors in the predicting QoL changes over time.

## Methods

### Design

This study was performed with the active collaboration of the French Association of Locked-in syndrome (ALIS). This is a non-profit association that was created in 1997 to help and support LIS patients and their families (http://alis-asso.fr/).

### Patients

The inclusion criteria were the following: adult patients, patients with LIS diagnoses according to the description of Plum and Posner [17] (i.e., complete or near-total loss of motor function, preservation of eye movements, anarthria, and preserved consciousness and intellectual function), and patients who agreed to participate. The exclusion criteria were the following: minors, and patients with major motor recuperation.

### Ethics

The study conformed to the principles of the Declaration of Helsinki and French Good Clinical Practices. According to the French law (Article L1121-1, Law n°2011-2012 29 December 2011 - art. 5), ethical approval is not needed. All subjects participated voluntary.

### Schedule

**Fig. 1:**
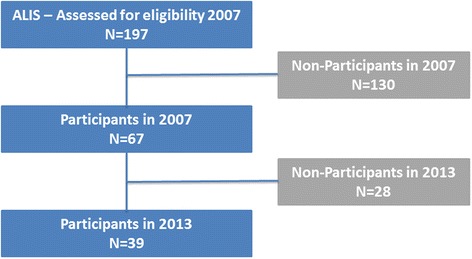
Flow chart

The contact information of the LIS patients was provided by the ALIS. Questionnaires were sent to 197 patients in 2007. The same questionnaire was sent again in 2013 to the 67 patients who responded in 2007. Thirty-nine (58 %) patients returned their questionnaires in 2013 (Fig. 1. Flow chart). This was 19.7 % of the original cohort in 2007. The patient could ask assistance to complete part or all of the questionnaire. Permission was granted by the ALIS to use the LIS patients’ data for the follow-up study.

### Data collection

The following data were recorded: i) sociodemographic data, including gender, age, living status, marital status, children, educational level, income, and religious status; ii) clinical data, including the type of LIS (total, classical, or partial) according to Bauer’s classification [18], disease duration, etiology (stroke, trauma, etc.), physical/handicap status (gastrostomy, tracheotomy, urinary probe, pain, wheelchair use, and communication ability, etc.), psychological status (presence of anxiety/depression and suicidal thoughts assessed from self-report items); iii) self-reported QoL as assessed with the Anamnestic Comparative Self-Assessment (ACSA), which provides an overall global assessment of the quality of life [19] based on the patient’s memories of the best period in their life before LIS and their worst period (ranging from −5 for the worst period and +5 for the best period; higher scores correspond to better QoL); and iv) Integration in life, i.e., the degree to which the patient has been able to return to a normal life as assessed using the French Reintegration to Normal Living Index (RNLI),[20, 21] which is an 11-item scale that covers areas such as participation in recreational and social activities and movement in the community. A 4-point Likert scale was used for this last test (no, mostly no, mostly yes, and yes) due to the specific constraint of minimal communication. The score was normalized to 100 (100 fully satisfied), and higher scores corresponded to better returns to normal life. The participants were also asked about end-of-life issues, i.e., the wish to be reanimated and willing for euthanasia.

The patients completed the self-report questionnaire directly through electronic communication devices or with the help of a caregiver.

### Statistical analysis

The continuous variables are expressed as the means, standard deviations, medians, and interquartile ranges (IQR). The qualitative variables are expressed as percentages. Nonparametric statistics were employed. The ACSA scores of the sample are described for 2007 and 2013. The mean ACSA scores were compared between 2007 and 2013 using the Wilcoxon signed rank test. A physical/handicap status change between 2007 and 2013 was defined as follows: a subject was been defined as exhibiting a change in his/her status when he/she required gastrostomy, tracheotomy, a urinary probe, and/or experienced chronic pain during the study period. The ACSA score in 2013 was tested with the following parameters using Mann–Whitney tests for qualitative variables and Spearman’s correlation coefficients for continuous variables: i) baseline parameters, ii) physical/handicap and psychological statuses in 2007, and iii) physical/handicap and psychological statuses in 2013. The ACSA score deltas between 2007 and 2013 were computed. Two groups of patients were defined: the patients with positive delta values corresponded to the QoL deterioration group, and the patients with negative or no delta values corresponded to the stable or improved QoL group. Comparisons between these 2 groups were performed according to the baseline general characteristics and the parameters of physical/handicap and psychological statuses in 2007. To determine variables linked to 2013 ACSA score, multivariate analyses using multiple linear regressions (forward-stepwise selection), were performed. The independent variables relevant to the models were selected from the univariate analysis, based on a threshold *p*-value of 0.10. The final models produced standardized beta coefficients, which represent a change in the SD of the dependent variable (ACSA score) resulting from a change of independent variables. Two models were performed: 1) a model including sociodemographics, LIS characteristics, and health status at 2007; 2) a model including sociodemographics, LIS characteristics, and health status at 2013.

The statistical analyses were performed using the SPSS software package, version 17.0 (SPSS Inc., Chicago, IL, USA). All tests were two-sided. Statistical significance was defined as *p* < 0.05.

## Results

### Sample in 2007

The main characteristics of the LIS patients are summarized in Table 1. A total of 67 patients returned their questionnaires in 2007. The sex ratio was 1.58 (males:females), and the mean age was 47 (SD 12). The median duration of LIS in 2007 was 7 years (IQR 4–9). The LIS etiology was stroke (ischemic or hemorrhagic) in 51 individuals. Regarding speech production, 77 % of the patients preferentially communicated via a communication board with a yes-no code, and 58 % had computer communication devices. Forty (61 %) individuals were autonomous with an electric wheelchair, half of these individuals had a gastrostomy, one-third had a tracheotomy, and 10 % had a permanent urinary probe. Forty-four percent of the patients suffered from chronic pain, 55 % had anxiety and/or mood disorders, and 27 % had suicidal thoughts. Sixty-seven percent envisaged resuscitation if needed, and two patients reported a wish for euthanasia.

**Table 1:** Characteristics of locked-in syndrome patients in 2007 and 2013

		Total of participants in 2007	Participants reporting QoL in 2007	Participants reporting QoL in 2013
		*N* = 67	*N* = 46	*N* = 39
1. Sociodemographics		N (%)	N (%)	N (%)
Gender	Woman	26 (39)	16 (35)	15 (39)
	Man	41 (61)	30 (65)	24 (62)
Age	Median [IQR]	47 [38;57]	51 [38;58]	51 [42;59]
Living status	Institutional setting	26 (39)	20 (44)	6 (18)
	Own home	41 (61)	26 (57)	27 (82)
Marital status	Couple	35 (57)	24 (57)	15 (45)
	Single	27 (43)	18 (43)	18 (55)
Children	No	24 (42)	16 (40)	15 (50)
	Yes	33 (58)	24 (60)	15 (50)
Educational level	< post-graduation	46 (71)	32 (70)	26 (67)
	≥ post- graduation	19 (29)	14 (30)	13 (33)
Month income (euros)	<1000	15 (27)	13 (33)	9 (36)
	≥1000	40 (73)	27 (68)	16 (64)
Religion	No religion	19 (29)	15 (33)	10 (74)
	Religious	46 (71)	31 (67)	29 (26)
2. LIS				
Type	Classical	47 (71)	31 (67)	20 (61)
	Partial	19 (29)	15 (33)	13 (39)
Disease duration	Median [IQR]	8 [4;9]	7 [4;9]	14 [10;15]
Etiology	Stroke	51 (82)	37 (84)	31 (82)
	Traumatic	8 (13)	5 (11)	4 (11)
	Others	3 (5)	3 (5)	3 (8)

*IQR* interquartile range

Forty-six patients (69 %) completed the QoL questionnaire. These patients did not differ from those who did not complete the QoL questionnaire in the main characteristics (i.e., sociodemographic and clinical data; all *p*-values > 0.05, data not shown). For these 46 patients, the median ACSA score was 1.5 (IQR [−3 to +3]).

### Change from 2007 to 2013

The main characteristics of the sample in 2013 are provided in Table 1. Two individuals died between 2007 to 2013. The 39 patients who returned their questionnaires in 2013, did not differ from the 28 who were lost to follow-up (i.e., gender, age, living status (2007), marital status (2007), children (2007), educational level, income (2007), and religious status (2007), type of LIS, disease duration, LIS etiology, all *p*-values > 0.05, data not shown). During the study period, of the 27 individuals who lived at home, one required institutionalization, and 6 of the 12 institutionalized patients in 2007 had returned home by 2013. Two of the 20 persons who were part of a couple in 2007 reported being single in 2013. None of the single individuals in 2007 were part of a couple in 2013.

**Fig. 2:**
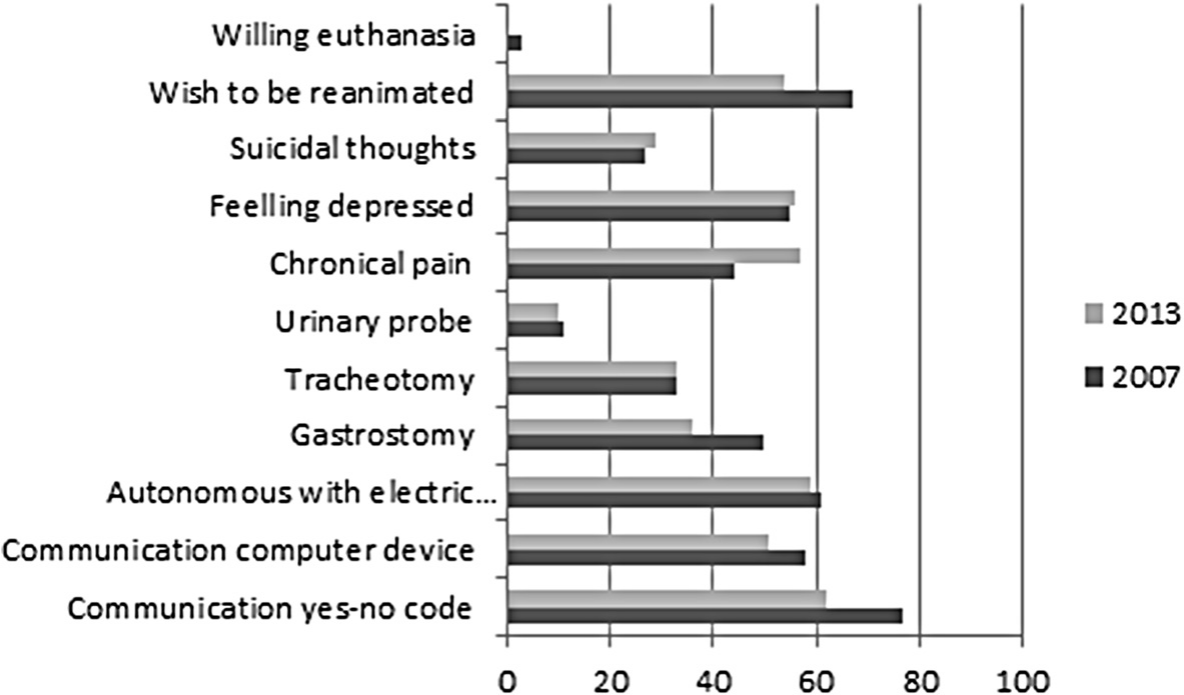
Change of physical/handicap and psychological statuses between 2007 and 2013

Among the 10 individuals without an electric wheelchair in 2007, only one acquired one during the 6-year period. Three patients with gastrostomies and tracheotomies in 2007 did not have them in 2013. Two (8 %) of 25 patients who did not have tracheotomies in 2007 had tracheotomies by 2013. Two (6 %) of the 36 patients who did not have urinary probes in 2007 had them by 2013. Eight of the 20 patients without chronic pain in 2007 exhibited it in 2013. Objective changes in physical/handicap statuses as defined by the need for a gastrostomy, tracheotomy, urinary probe, or the occurrence of chronic pain were noted in 11 (28 %) of the 39 patients. Thirty-one percent of the patients developed anxiety or mood disorders during the study period, and 3 individuals reported new suicidal ideas. These findings are detailed in Fig. 2. The person who wished for euthanasia in 2007 reported not wishing for euthanasia in 2013. Of the 23 subjects who wanted resuscitation if needed in 2007, only 15 of maintained this prerogative in 2013.

### Factors associated to QoL change over time

Thirty-nine (58 %) patients returned their questionnaires in 2013. The median ACSA score of these patients was 3.0 (IQR 0–3). Both 2 QoL scores (2007 and 2013) were only available for 30 individuals. These 30 patients did not differ from the 16 patients who were lost to follow-up (all *p*-values > 0.05, data not shown). While QoL did not statistically differ between the 2 periods (*p* > 0.05), 21 (70 %) of the individuals reported a stable (*n* = 10) or improved (*n* = 11) QoL between 2007 and 2013 according to the ACSA scores.

The QoL at 6 years was not significantly related to the baseline sociodemographic characteristics of the patients. No significant differences were found in terms of gender, age, living status, marital status, having children, educational level, income, or religious status. No statistical relationships of QoL with disease duration, LIS type, or LIS etiology were found. The physical/handicap statuses in 2007 were not related to the QoL 6 years later; having a gastrostomy, tracheotomy, or permanent urinary probe or the presence of chronic pain were not linked to QoL in 2013. The individuals who reported the use of the yes-no code reported a significantly lower QoL in 2013 compared to the non-users, and the individuals with electric wheelchairs tended to report higher QoL scores in 2013 compared to those without electric wheelchairs (*p* = 0.07). Similarly, no indicators of the physical/handicap statuses in 2013 were statistically related to the 2013 QoL score with the exception of one communication parameter, i.e., the individuals who used yes-no code reported significantly lower QoL levels than those who did not (*p* = 0.02). The patients with suicidal ideas reported significant lower QoL levels than those without such thoughts (0.5 ± 3.1 versus. 2.0 ± 2.4, *p* < 0.008). All of the relevant details (variables with a *p*-value ≤0.20) are reported in Table 2. No differences in baseline characteristics (i.e., sociodemographic, LIS, and health/handicap status characteristics) were found between the individuals who exhibited QoL deterioration during the 6-year period and the individuals who exhibited with stable/improved QoL (data not shown). The QoL levels in 2013 did not differ between the subjects who exhibited objective deteriorations in physical/handicap statuses (as defined by new needs for gastrostomy, tracheotomy, a urinary probe, and/or the new occurrence of chronic pain between 2007 and 2013) and the subjects who exhibited stable or improved physical/handicap statuses (0.8 ± 3.2 versus 1.6 ± 2.7, *p* = 0.436).

**Table 2:** Anamnestic Comparative Self-Assessment score in accordance with sociodemographic and clinical variables^*^ (*N* = 39)

		ACSA score 2013^a^	
		Med [IQR]	*p*
1. LIS			
Disease duration	Correlation	*R* = 0.260	0.110
2. Physical/handicap status in 2007			
Permanent urinary probe	No	3.0 [0;3.0]	0.119
	Yes	−2.0 [−5;2]	
Autonomous with electric weelchair	No	−0.5 [−4.2;2.8]	0.065
	Yes	3.0 [0;3.0]	
Use yes-no code	No	3.0 [3.0;4.0]	0.023
	Yes	2.0 [−1.8;3.0]	
3. Psychological status in 2007			
Suicidal thoughts	No	3.0 [0;4.0]	0.109
	Yes	1.5 [−2.0;3.0]	
4. Physical/handicap status in 2013			
Use yes-no code	No	3.0 [2.3;4.0]	0.021
	Yes	2.0 [−2.0;3.0]	
Electronic communication device	No	3.0 [1.0;3.5]	0.123
	Yes	1.5 [−2.0;3.0]	
5. Psychological status in 2013			
Suicidal thoughts	No	3.0 [0.8;4.0]	0.008
	Yes	0 [−5.0;2.3]	
RNLI	Correlation	*R* = 0.283	0.116

*ACSA* anamnestic comparative self-assessment

*RNLI* reintegration to normal living index, higher scores indicate better return-to-normal-life (range [0–100])

*Med [IQR]* median [interquartile range]

^*^Only variables with a *p*-value ≤0.20 were presented

^a^Higher score indicates higher QoL (range [−5;+5])

According to the predefined rules (statistical section), two multivariate models were performed. In the first model, autonomous electric chair in 2007 (*p* = 0.065) and use yes-no code in 2007 (*p* = 0.023) were selected and neither was significantly linked to the ACSA score at 2013 after adjustment. In the second model, use yes-no code in 2013 (*p* = 0.021) and suicidal thoughts in 2013 (*p* = 0.008) were selected and only suicidal thoughts is significantly associated with ACSA QoL score at 2013 after adjustment (*β* = −0.457, *p* = 0.006).

## Discussion

This is the first study to report on the evolution of QoL in a LIS population and is the largest and longest duration examination of a cohort of LIS patients ever conducted.

The LIS persons involved in this study already had chronic LIS statuses for long durations (the median LIS duration was 7 years in 2007). Several previous studies have reported the QoL of LIS persons in single assessments [14, 22, 23] through cross-sectional research studies that may not provide definite information about cause and effect relationships. Longitudinal design provides more valid information and more robust findings.

The first important finding is that the LIS patients exhibited a rather good QoL maintenance throughout the 6-year period; nearly ¾ of the patients reported a stable or improved QoL at 6 years. Regarding the few studies that have used the ACSA score to assess QoL, LIS patients have reported lower scores (after scoring harmonization) than subjects who have sustained whiplash injuries [24] and higher scores than patients with facial prostheses [25] and patients with new diagnoses of Alzheimer’s disease [26].

In the same period, the health/handicap (physical and psychological) statuses of some of the patients changed. We did not observed relationships between the objective parameters of the health/handicap statuses and level of QoL. Similarly, considering the 11 patients (of the total of 39) who objectively exhibited deteriorations in their health/handicap statuses between 2007, their QoL scores were not different from those of the other patients. It has been previously observed that patients’ subjective QoL is not related to physical impairments; this observation agrees with previous studies of different motor neuron disorders [4, 11, 15, 22, 27–32] and illustrates the “disability paradox” reported by Albrecht and Devlieger [33]. These findings confirm that usual clinical assessments that are based on objective outcomes do not reflect all of the aspects that patients consider important in to live. This lack of association between objective health/handicap change and QoL could also be explained by the presence of the well-known ‘response shift phenomena’ [34]. QoL is self-reported by the patient and might be influenced by this phenomena, which corresponds to the adaptation to the illness (i.e., adaptation to a bedridden state and restricted physical/social function in the specific case of LIS). The presence of a response shift may result in the over- or underestimation of the true changes and lead to challenges in interpreting QoL measures, especially in longitudinal studies [35]. In this present case, the three classical components of the response shift may have been incriminated; i.e., reconceptualization defined by as a redefinition of QoL, reprioritization defined as a change in the importance attributed to the component domains that constitute QoL, and recalibration defined as a change in a patient’s internal measurement standard.

Although medical device use declined over time in terms of feeding tubes and tracheotomies, an important proportion of patients continued to depend on these devices throughout the study period. Indeed, in 2013, a high proportion of the patients (one-third) had gastrostomies and tracheotomies primarily due to incomplete swallow recovery, and 10 % had permanent urinary probes. In most studies, swallowing ability and improved continence have been found to reduce the need for medical devices [3, 4].

Another interesting finding concerns the role of communication. All of the LIS persons involved in this study could communicate; more than 50 % of the patients used electronic communication devices, and the other patients communicated only through a yes-no code. The proportion of patients who preferentially used a yes-no code to communicate tended to decrease over time, from 77 % in 2007 to and 62 % in 2013. This last restrictive mode of communication was the single parameter that was associated with a significantly lower QoL. Communication for LIS people implies the use of alternative communication, such as eye blinks or eye movements for a yes-no code or communication boards with letters or symbols that are indicated via eye movements, and both alternatives imply the avoidance of open-ended and the confirmation of answers with repeated questions when necessary. Communication is also very limited and requires the help of others. Electronic communication devices, including patient-computer interfaces such as infrared eye movement sensors and computer voice prosthetics, have a liberating effect on people with LIS and enable them to have real dialogues and use the internet instead of passively responding to the requests of others [36–38].

The autonomy afforded by an electric wheelchair is recognized as an important element for an LIS patient. In our study, nearly 60 % of the LIS patients stated that they were autonomous with a powered electric wheelchair and reported feelings of sufficient autonomy at home, and such feelings were associated with higher QoL scores, although this difference was not significant.

Another comment should be made concerning the life conditions of the patients. In 2013, 82 % (32/39) of the patients lived in their own homes. Among these patients, 81 % were already at home in 2007, but 19 % moved from an institutional setting to their personal home, which implies that both the health/handicap status and the family circle allowed for such a move. Surprisingly, the patients living in institutional settings reported systematically higher scores than those living at home (albeit this difference was non-significant), which could be explained by the soothing role of a medical environment. Marital status did not affect the QoL score, but single persons generally reported lower scores than the individuals who were part of a couple. Financial income was not related to overall well-being, which is probably reflective of the French health care system, specifically the universal health-care insurance and existence of resource allocations to help maintain very dependent persons at home via the financing of human and technical aid.

The mental/psychological conditions are important to consider. We did not observe a link between mood disorders and QoL levels, although a high proportion of our patients reported having mood disorders and/or feelings of depression. Previous studies that have assessed patients with severe diseases (e.g., amyotrophic lateral sclerosis) have reported contradictory relationships between depression and QoL [39]. Nevertheless, more than a quarter of our cohort reporting having suicidal thoughts as previously described,[40] and such thoughts unsurprisingly influence their QoL. Lastly, family members, careers and medical professionals frequently assume that LIS persons would choose to die, but this was not true. Our results indicate that the demand for euthanasia was almost non-existent in our group of patients, and a great number of the patients expressed a desire for resuscitation if necessary.

### Strengths and limitations

The sample size was arguably too small. When we tried to identify linked factors using the multivariate approach, associations may have been missed due to low statistical power. Larger samples would allow for the confirmation of these findings despite the rarity of this condition. However, the present report is the only study that has followed patients for a rather long period.

The representativeness and the size of our sample should be discussed. Our patients appeared to be relatively similar to the populations of other studies in terms of age, gender, and LIS etiology [3, 4, 41]. As described in previous studies, [4, 5] the mortality rate was very low; two patients died in 6 years after initial medical stabilization (more than a year). According to Doble et al., [4] the 10-year survival of LIS persons is 83 %, and the 20-year survival is 40 %. The limited data preclude an appreciation of the true prevalence/incidence of this syndrome, [38]. While it can be assumed that a significant proportion of individuals with LIS are in contact with the French Association of Locked-In Syndrome, we can hypothesize that these individuals have a better social support compared to the individuals who are not in contact with patients’ associations. Future studies should better apprehend the exhaustiveness.

The proportion of patients lost to follow-up appears high and troublesome to the significance of findings. It could be hypothesized that the non-respondents included patients with more severe physical and/or mental and/or social conditions, more severe communication limitations, and more important cognitive impairment. This can lead to an overestimation of the QoL level. However, we can assume that the respondents did not differ from the non-respondents in terms of gender and age, which ensures the relative validity of our findings.

We were unable to confirm the impact of other potential QoL determinants. Cognitive impairment, social support and social bonding, and satisfaction with these supports were not collected in our study. Future studies should explore these parameters.

Finally, the communication limitations of LIS persons make patients’ assessments particularly difficult. As we did not collect the information whether the patient him/herself responded or if the caregiver filled out the questionnaire on behalf of the patient, we can provide an inaccurate estimation of the patient’s QoL. The QoL scale had to be selected based on the ease of use with this population and consisted mostly of the use of eye blinking and vertical eye movements to communicate. The use of the self-report Anamnestic Comparative Self-Assessment to determine the QoL level should be discussed. Indeed this questionnaire provides a single global measure restricting the QoL concept. Although we appreciate this tool for its capacity for self-administration and its short time of completion, we recognize that a more specific QoL questionnaire or a multidimensional questionnaire could provide a more satisfactory picture of the self-perceived lives of these patients.

## Conclusion

This is the first study to assessing QoL changes over time in a LIS population. The main message of this work is that LIS persons report relatively satisfactory QoL levels that are stable over time. These findings suggest that life with LIS is worth living in contrast to the general and widespread opinions. The preservation of communication likely help LIS patients live as normally as possible. Replication of these findings in larger groups of patients is required.

## Abbreviations

ACSA: Anamnestic Comparative Self-Assessment
ALIS: Association of locked-in syndrome
LIS: Locked-in syndrome
QoL: Quality of life
RNLI: Reintegration to Normal Living Index
